# Letter from the New Editor-in-Chief

**DOI:** 10.3390/life4010001

**Published:** 2014-01-08

**Authors:** Pabulo Henrique Rampelotto

**Affiliations:** Interdisciplinary Center for Biotechnology Research, Federal University of Pampa, Antônio Trilha Avenue, P.O. Box 1847, 97300-000, São Gabriel–RS, Brazil; E-Mail: pabulo@lacesm.ufsm.br

It is my great pleasure to serve as the new Editor-in-Chief of *Life*, a journal concerned with fundamental questions on the origins and nature of life, evolution of biosystems and astrobiology.

With my experience as Executive Editor, Senior Editor and Guest Editor of so many successful special issues (some of them in MDPI journals [1,2,3,4,5,6]), I am committed to making the journal a success, with the launch of exciting special issues, publication of high quality papers, as well as inclusion of the journal in major indexing and abstracting services.

I see *Life* as an exciting interdisciplinary open access journal that will grow to become a valuable, widely read and widely cited part of the scientific literature. The journal has the potential to be internationally recognized for its quality and the scientific influence of its contributions, achieving high reputation among the scientific community in the coming future.

My vision for the direction and progress of this journal is that it should cover not only the classical themes, but also other frontier research topics. This journal will foster fruitful crosstalk between the various traditional and novel disciplines of life sciences and related fields of research, including physical and chemical aspects that may help us to better understand the origins of life and evolution of biosystems.

As an enthusiast on theoretical and philosophical studies involving life sciences, I would like very much to have more papers in these fields of knowledge published in *Life*. In addition to experimental studies, *Life* provides a forum for the publication of new hypotheses aiming to encourage discussion and creative hypothesis testing by members of the scientific community. The predictions of the hypothesis must be amenable to further observation and experimentation that could tend to confirm or refute the hypothesis. 

An excellent example in this matter is the classical work published by Watson and Crick in 1953, where they used data published by other scientists to speculate on a new structural model for the DNA molecule [7]. Considering the current state of the reviewing process of most journals, if they had submitted their proposal today, the paper would be rejected or reconsidered after major revision and they would be advised to include some new experimental data to support their model. Nevertheless, at that time, the paper was quickly published and was indeed validated by future experiments. Today, that paper is considered one of the main landmarks in science, giving birth to molecular biology. Suddenly, a paradigm was created that gave the study of heritable traits a physical, molecular basis and made the theories of Mendel, Morgan and even Darwin, tangible.

Instead of trying to predict if a theoretical study will be proven right or wrong or trying to predict the future impact of an experimental study, our focus on reviewing papers for consideration and possible publication will be on determining if the work is scientifically well written and presents coherent arguments. In 1974, Francis Crick, reflecting on the impact of the publication of his work on the structure of DNA that won him the Nobel Prize, suggested that it would be for historians to decide the impact of his work [8]. 

A high quality study, even when further proved to be incomplete or mistaken, may play a relevant role in the scientific process, by allowing others to test the ideas and build on new experiments/hypotheses that will help us to better understand some particular aspect of nature. An excellent example comes from another landmark paper also published in 1953. The classic experiment of Stanley Miller was the first experimental evidence that the hypothesis of Oparin-Haldane (which postulates that the first living systems in our planet are the result of a long chemical evolution) could be correct. In the experiment, Miller demonstrated in laboratory, the formation of organic compounds of biological interest in conditions similar to the primitive atmosphere [9]. Today, the experiment of Miller is not anymore considered representative of the chemical processes that occurred on Earth billions of years ago because his model of primitive atmosphere does not fit with the current model. However, with such a pioneering experiment, Miller began the development of the experimental research on chemical evolution and the origins of life. For this achievement, Miller is considered the founder of what is now known as prebiotic chemistry, the exciting field of research that investigates the chemical reactions that lead or could have led to the emergence of life on our planet or elsewhere in the Universe.

For these reasons, a new journal such as *Life*, with an open-minded perspective capable of dealing with genuinely innovative science, is necessary because recent advances in different fields of life sciences is fundamentally changing how we think about the origins and evolution of organisms. Scientists need a high quality journal where these cutting edge experimental and theoretical studies can be quickly published and divulgated to the scientific community. Furthermore, the whole field of life sciences is expanding quite fast and the result is that good quality journals are receiving increasing numbers of submissions, resulting in slow processing times for reviewing and publication. In addition, many perfectly sound papers are rejected because they do not fit within the limited scope of a journal or due to the limited number of papers published in a printed journal.

At a time when publication in major journals can take several months after a paper is accepted and can cost a significant portion of a laboratory’s research budget, the open-access format of *Life* provides a platform for fast and cost-effective publication without undue delay or expense. All submitted manuscripts undergo rigorous peer review, but we ensure that this is done as fast as possible because we endeavor to provide an efficient reviewing process for the authors.

*Life* is currently supported by an outstanding Editorial Board composed of eminent team leaders who cover the wide remit of the journal. In cooperation with the editorial board members and a dedicated editorial office, I will make every effort to continue the progress of *Life* in a manner that will satisfy our authors and readers. To promote the development of the journal, we are planning an ambitious series of special issues devoted to topics of particular interest and importance in life sciences and related disciplines. This is an exciting moment for *Life* and I welcome you to submit your manuscript and enjoy a pleasant experience while working with our editorial staff.
